# Effects of suspension exercise training in the treatment of lumbar disk herniation: a systematic review and meta-analysis

**DOI:** 10.3389/fneur.2024.1455505

**Published:** 2024-12-02

**Authors:** Yu’ang Liu, Silang Huang, Xinxin Zhang, Huangying Liao, Weiguo Liu, Zhi Zhang

**Affiliations:** ^1^College of Physical Education and Health, Guangxi Normal University, Guilin, Guangxi, China; ^2^College of Education, Guilin University, Guilin, Guangxi, China

**Keywords:** suspension exercise training, lumbar disc herniation, pain, Meta-analysis, review

## Abstract

**Objective:**

The study aimed to systematically evaluate the efficacy of suspension exercise training (SET) in the treatment of lumbar intervertebral disk herniation and provide a scientific basis for clinical treatment.

**Methods:**

Databases such as CNKI, Chinese Wanfang, PubMed, Cochrane, the Web of Science, and Embase were searched up to June 2024. A quality assessment was performed using the Cochrane Collaboration’s risk-of-bias guidelines, and a meta-analysis was conducted using RevMan 5.4 and Stata 17.0.

**Results:**

A total of 11 studies involving 943 patients were included. Suspension exercise training significantly improved the lumbar disk herniation (LDH) visual analog scale (VAS) score (mean difference (MD) = −0.96; 95% confidence interval (CI), −1.10 to-0.82; *p* < 0.00001, *I^2^* = 23%), the Japanese Orthopedic Association (JOA) score (MD = 3.29, 95% CI, 1.67 to 4.90; *p* < 0.0001, *I^2^* = 92%), and the Oswestry Disability Index (ODI) score (MD = −5.41, 95% CI, −7.41 to −3.40; *p* < 0.00001, *I^2^* = 86%). Subgroup analysis of the JOA score showed better efficacy with suspension exercise training combined with traditional Chinese medicine (TCM) (MD = 4.29, 95% CI, 2.73 to 5.86; *p* < 0.00001, *I^2^* = 80%) compared to suspension exercise training combined with non-TCM (MD = 0.96, 95% CI, 0.49 to 1.43; *p* < 0.0001, *I^2^* = 0%).

**Conclusion:**

Suspension exercise training significantly improved the VAS score, JOA score, and ODI score of the patients with lumbar disk herniation; however, there was a high degree of heterogeneity in the JOA score and ODI score. Further validation is needed in the future for different populations with lumbar disk herniation, the specific locations of its occurrence, and the combined modality of suspension exercise training.

**Systematic review registration:**

https://www.crd.york.ac.uk/prospero/, identifier CRD42024554074.

## 1 Introduction

Lumbar disk herniation (LDH) is a common clinical spinal disorder in which the fibrous annulus of the lumbar disk partially or completely ruptures due to various causes. This rupture causes the nucleus pulposus tissue to protrude backward, irritating or compressing the nerve roots and cauda equina (1). Low back pain and neurological dysfunction are the main clinical manifestations (2). In recent years, the incidence of LDH has been increasing annually and shows a trend toward younger age groups (3). It is estimated that approximately 2–3% of the world’s population experiences LDH (4), which mainly affects adults between the ages of 20 and 50 years (3). Intensive work, long hours, sedentary behavior, and prolonged standing are considered the main causes of LDH. Difficulties in standing, walking, and performing simple tasks in patients with LDH have significantly impacted global productivity, public health, and the quality of life for those affected (5, 6). The United States spent up to $4 billion on treating LDH through medication and surgery in 2015 alone (7).

The treatment of LDH can be broadly categorized into surgical and non-surgical approaches. Although surgical treatments can provide rapid pain relief, they are associated with significant drawbacks, such as postoperative complications, technical challenges, a high risk of reoperation, and substantial costs (8–10). Consequently, 80–85% of patients opt for non-surgical treatments (11).

Non-surgical treatment options for LDH include a variety of therapeutic approaches aimed at alleviating symptoms and improving function without the need for invasive procedures. These options include physical therapy, pharmacological treatments, chiropractic care, and acupuncture (12–14). Suspension exercise training (SET) is emerging as a promising non-invasive therapy for treating skeletal and muscular disorders, including LDH. SET is simple, easy to perform, painless, relatively inexpensive, and effective (10). Despite its potential benefits, there has been no comprehensive systematic review or meta-analysis specifically evaluating the efficacy of SET for treating LDH. Our study aimed to provide a new option for the treatment of LDH.

## 2 Methods

This study was registered in PROSPERO (CRD42024554074) and strictly adhered to the Preferred Reporting Items for Systematic Reviews and Meta-Analyses (PRISMA) statement (15).

### 2.1 Literature search and selection

We searched the CNKI, Chinese Wanfang, PubMed, Cochrane, Web of Science, and Embase databases for Chinese and English literature up to June 2024. The search terms included “sling exercise, ““suspension exercise,” and “LDH.” The inclusion criteria were as follows: (1) treatment involving suspension exercise; (2) patients diagnosed with LDH; (3) study design as a randomized controlled trial or a clinical trial; and (4) the visual analog scale (VAS) score, Japanese Orthopedic Association (JOA) score, and Oswestry Disability Index (ODI) score of suspension exercise for LDH. The exclusion criteria were as follows: (1) no control group in the trial; (2) no data on baseline or endpoint outcomes; (3) patients with psychiatric disorders; and (4) reviews, dissertations, or conference papers.

Duplicates were removed using EndNote X9 software. Two authors (SL and XX) independently read the titles and abstracts of the literature to determine whether the inclusion criteria were met. The studies that initially met the inclusion criteria were read in full to determine final inclusion. For disagreements regarding the studies, a third author (HY) was involved to help determine inclusion through discussion.

### 2.2 Data extraction and quality assessment

Data extraction from the final included studies was performed independently by two authors (SL and HY), and the extracted information included the first author, year of publication, basic information about the participants, types of interventions, intervention duration, outcome indicators, and follow-up time.

The Cochrane Collaboration’s risk-of-bias guidelines (16) were used to evaluate the quality of the included studies. The guidelines included the following: (1) random sequence generation, (2) allocation concealment, (3) blinding of participants and personnel, (4) blinding of outcome assessments, (5) incomplete outcome data, (6) selective reporting, and (7) other bias. The quality of the studies was evaluated independently by two authors. In case of a disagreement, a third author was involved in discussions until an agreement was reached.

### 2.3 Types of outcome indicators

The primary outcome indicators included the visual analog scale (VAS) (17), and the secondary outcome indicators included the Japanese Orthopedic Association (JOA) (18) and Oswestry Disability Index (ODI) (19).

### 2.4 Data synthesis and statistical analysis

All outcome indicators in this study were continuous variables measured on the same rating scale and were analyzed using mean difference (MD) and a 95% confidence interval (CI) as effect sizes. The effect size resulting from the meta-analysis provided a statistically standardized representation of the quantitative results of each study. It was calculated based on the mean pre-post change in the experimental group minus the mean pre-post change in the comparison group and then divided by the pooled pretest standard deviation. The meta-analysis was performed using RevMan 5.4 and Stata 17.0. Heterogeneity was quantified using the *I^2^* statistic, and a fixed effects model was employed if the difference in the heterogeneity test was not statistically significant (*I^2^* < 50%; *p* > 0.05). Otherwise, a random effects model was applied. Subgroup and sensitivity analyses were performed to explore the sources of heterogeneity for the outcome indicators. The subgroup analyses were performed according to the type of interventions, while the sensitivity analyses were performed by removing each study item to assess the reliability and consistency of the results. Publication bias was assessed using funnel plots and Egger’s asymmetry test for outcome measures with more than 10 included studies. All statistical significance levels were set at *α* = 0.05.

## 3 Results

### 3.1 Search results

**Figure 1:**
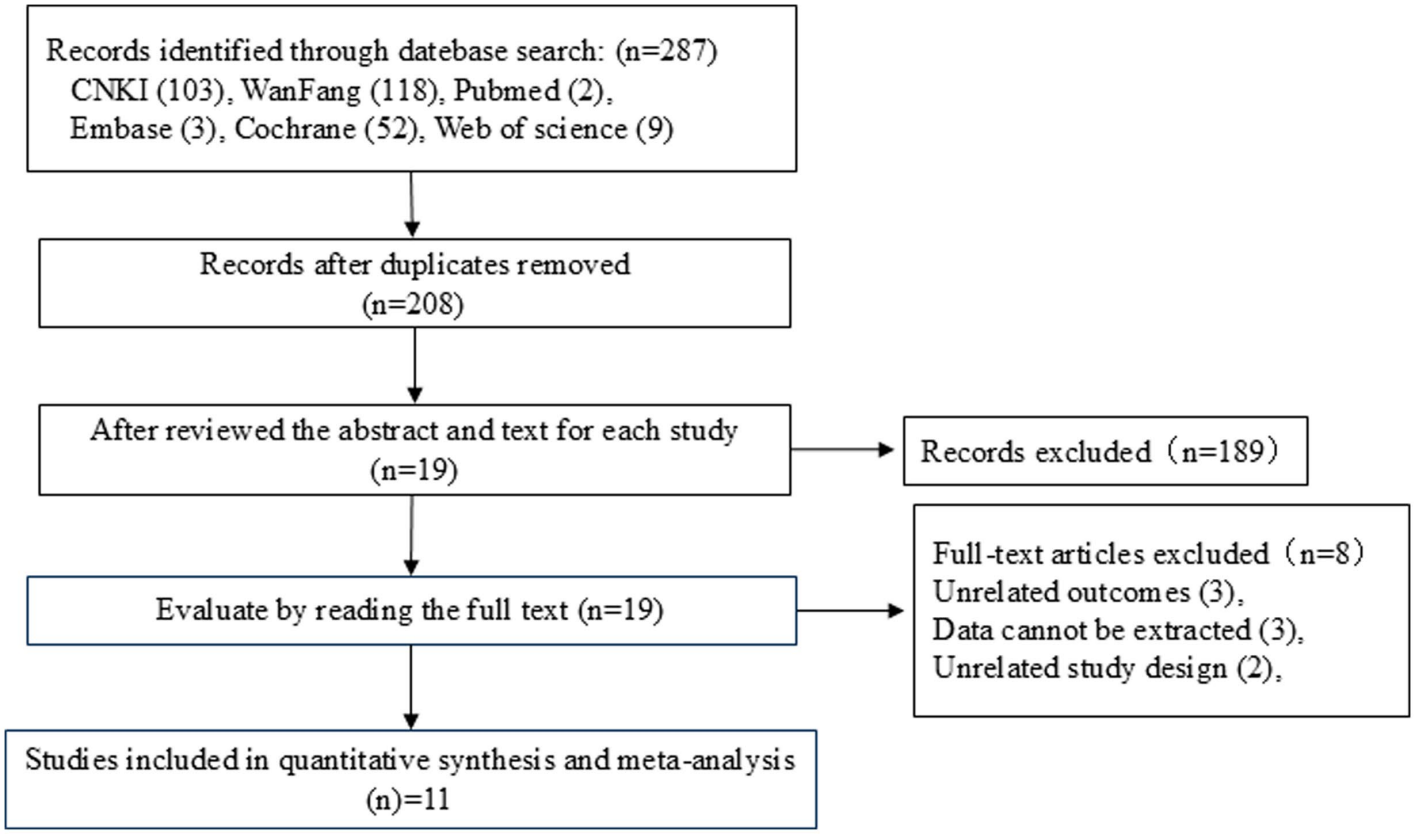
Literature screening flowchart.

A total of 287 studies were identified through database searches, and 208 studies remained after removing duplicates using EndNote X9. Two authors reviewed the titles and abstracts of the studies according to the inclusion and exclusion criteria for preliminary screening. In case of a disagreement between the two authors, a third author was involved in the discussion and decided whether to include a study. A total of 189 irrelevant studies were excluded, leaving 19 articles that were read in full. An additional eight articles that did not meet the inclusion criteria were excluded, resulting in a final total of 11 articles that were included in the meta-analysis. The literature screening process is shown in Figure 1.

### 3.2 Basic characteristics of the included studies

This meta-analysis included 11 studies (20–30), involving 943 patients—472 in the test group and 471 in the control group. There were 463 male participants and 397 female participants, with two studies not reporting the sex of the participants. The mean age of the patients ranged from 37.2 to 58.6 years, with one study not reporting the mean age. The intervention period ranged from 2 to 8 weeks. The outcome indicators included the VAS, JOA, and ODI scores. Six studies reported follow-up, while five did not (see Table 1).

**Table 1:** Basic characteristics of the included studies.

Reference	Age (Mean ± SD)	Sex (M/F)	Sample size	Intervention type	Intervention period	Outcome indicators	Follow-up
Yang et al. (29)	E: 58.6 ± 10.5	E: 17/23	E: 40	SET + McGill	2 weeks	①②③	Yes
	C: 55.0 ± 9.4	C: 11/29	C: 40				
Sun et al. (27)	E: 47.5 ± 11.3	E: 15/15	E: 30	SET + Rehabilitation	2 weeks	①	Yes
	C: 46.7 ± 10.9	C: 14/16	C: 30				
Du et al. (21)	E: 47.4 ± 14.6	E: 18/14	E: 32	SET + Breathing training	2 weeks	①②	Yes
	C: 48.2 ± 15.8	C: 17/15	C: 32				
Ding et al. (20)	E: 43.6 ± 8.8	E: 15/13	E: 28	SET + Tuina	4 weeks	①②	Yes
	C: 44.1 ± 8.5	C: 13/15	C: 28				
Li et al. (23)	E: 42.8 ± 11.1	E: 15/15	E: 30	SET + Massage	4 weeks	①②③	No
	C: 43.0 ± 10.8	C: 19/11	C: 30				
Li et al. (24)	E: 43.6 ± 10.2	E: 33/26	E: 59	SET + Massage	4 weeks	①②	No
	C: 43.1 ± 10.2	C: 31/28	C: 59				
Liang et al. (26)	NR	NR	E: 29	SET + Rehabilitation	4 weeks	①③	No
	NR	NR	C: 29				
Zhang et al. (30)	E: 53.0 ± 5.1	E: 16/14	E: 30	SET + Shockwave training	4 weeks	①③	Yes
	C: 52.9 ± 5.1	C: 15/15	C: 30				
Li et al. (25)	E: 46.7 ± 10.2	E: 17/15	E: 32	SET + Rehabilitation	4 weeks	①②	Yes
	C: 47.3 ± 9.2	C: 17/13	C: 30				
Khanzadeh et al. (22)	E: 37.2 ± 5.3	NR	E: 12	SET + core stability exercises	8 weeks	①	No
	C: 43.4 ± 8.6	NR	C: 13				
Xue et al. (28)	E: 56.5 ± 5.4	E: 91/59	E: 150	SET + pregabalin	4 weeks	①②③	No
	C: 56.4 ± 5.6	C: 89/61	C: 150				

E, experimental group; C, control group; M, male; F, female; NR, not reported; and SET, suspension exercise training.

### 3.3 Quality evaluation results

**Figure 2:**
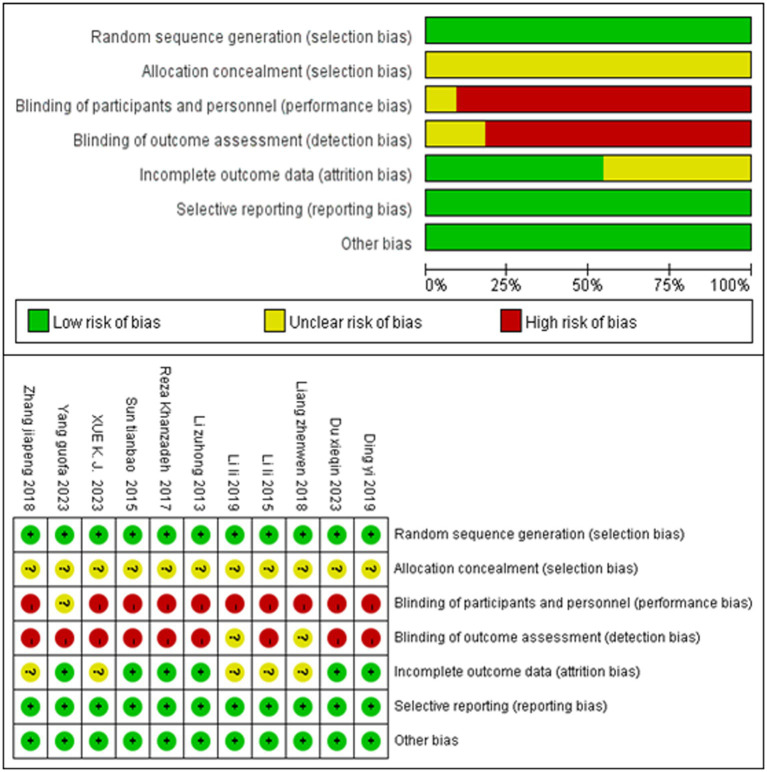
Quality evaluation results.

This meta-analysis used the Cochrane Collaboration’s risk-of-bias guidelines to assess the quality of the included studies. Regarding random sequence generation, 11 studies were assessed as low risk because they all reported random allocation using the random number expression method. Regarding allocation concealment, 11 studies reported that the method used for allocation concealment was not clearly stated, and thus they were assessed as uncertain risk. In terms of blinding of participants and personnel, one study that indicated that the intervention was conducted uniformly was assessed as uncertain risk, while 10 studies that did not report implementation blinding were assessed as high risk. Regarding blinding of outcome assessments, two studies that reported that the blinding of outcome assessments was performed by uniform professionals were assessed as uncertain risk, while nine studies that did not report on the blinding of outcome assessment were assessed as high risk. In terms of incomplete outcome data, six studies with complete outcome data were assessed as low risk, while five studies were assessed as uncertain risk for not reporting follow-up. In terms of selective reporting, all 11 studies that reported findings were assessed as low risk. In terms of other biases, all 11 studies were assessed as low risk, with no additional biases identified. The results of the quality assessment are shown in Figure 2.

### 3.4 Meta-analysis results

#### 3.4.1 Meta-analysis of SET on the VAS scores

**Figure 3:**
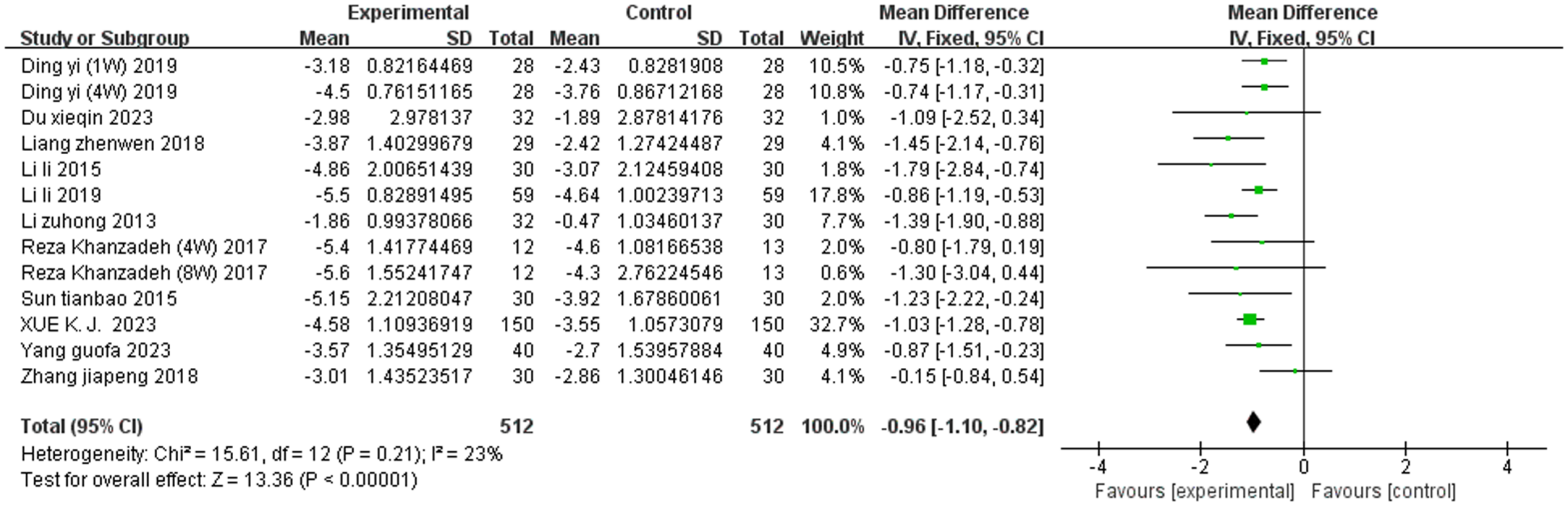
Meta-analysis of SET on the VAS score.

Two studies assessed the VAS scores at different time points; therefore, a total of 13 VAS score comparisons were reported across 11 studies. A fixed effects model was used to integrate the results. SET significantly reduced the VAS score among the patients with LDH compared to the controls (MD = −0.96, 95% CI, −1.10 to −0.82; *p* < 0.00001, *I^2^* = 23%) (Figure 3).

#### 3.4.2 Meta-analysis of SET on the JOA scores

**Figure 4:**
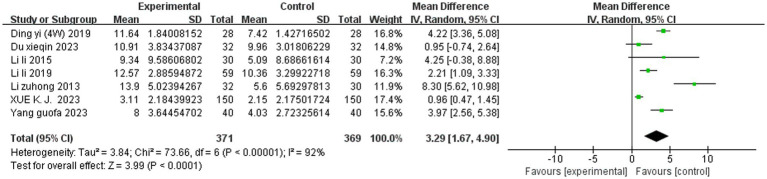
Meta-analysis of SET on the JOA score.

**Figure 5:**
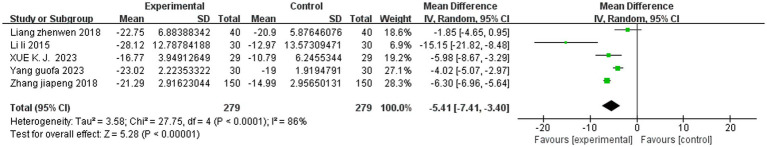
Subgroup analysis of SET on the JOA score.

Seven studies reported the JOA scores. A random effects model was used to integrate the results. SET significantly improved the JOA score among the patients with LDH compared to the controls (MD = 3.29, 95% CI, 1.67 to 4.90; *p* < 0.0001, *I^2^* = 92%). Subgroup analyses of the JOA score based on intervention modality revealed that SET combined with traditional Chinese medicine (TCM) (MD = 4.29, 95% CI, 2.73 to 5.86; *p* < 0.00001, *I^2^* = 80%)had better efficacy compared to SET combined with non-TCM (MD = 0.96, 95% CI, 0.49 to 1.43; *p* < 0.0001, *I^2^* = 0%) and that intervention modality was the main source of heterogeneity in the JOA score among the patients with LDH (Figures 4, 5).

#### 3.4.3 Meta-analysis of SET on the ODI scores

**Figure 6:**
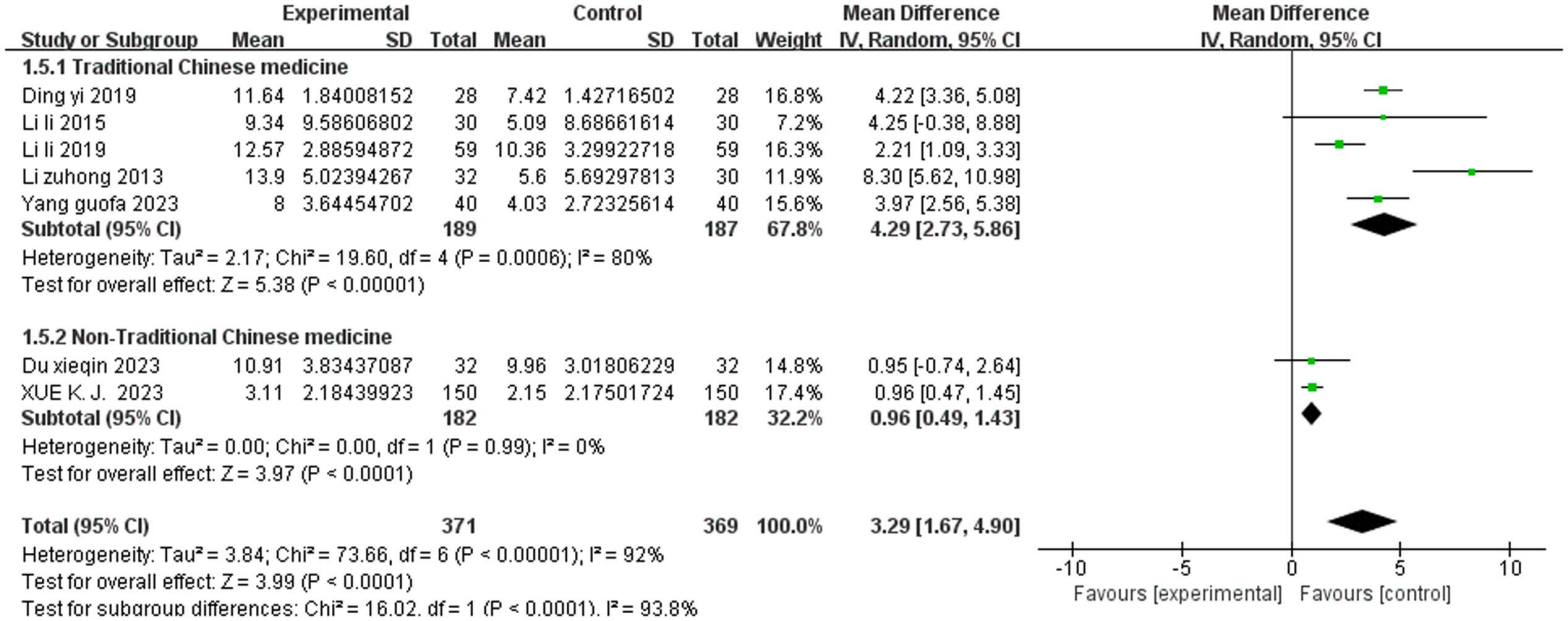
Meta-analysis of SET on the ODI score.

Five studies reported the ODI scores. A random effects model was used to integrate the results. SET significantly improved the ODI score among the patients with LDH compared to the controls (MD = −5.41, 95% CI, −7.41 to −3.40, *p* < 0.00001, *I^2^* = 86%). Subgroup analyses based on the intervention period and intervention type did not reveal any sources of heterogeneity (Figure 6).

### 3.5 Sensitivity analysis

Heterogeneity for the two outcome indicators, the JOA score and ODI score, was high in this study. By removing one JOA score and one ODI score (whose treatments in this study were different from those in the other studies) through sensitivity analysis, the remaining combined results showed a significant decrease in heterogeneity (*I^2^* = 83% and *I^2^* = 80%). However, there was still no statistically significant difference in the total combined results for the JOA scores (MD = 3.73, 95% CI, 2.17 to 5.19, *p* < 0.00001) and ODI scores (MD = −5.37, 95% CI, −8.33 to −2.42, *p* < 0.0004), indicating that the findings of this study are relatively reliable. The detailed sensitivity analyses for the VAS score, JOA score, and ODI score are presented in Supplementary Tables S2–S4 of the attachment, respectively.

### 3.6 Publication bias analysis

**Figure 7:**
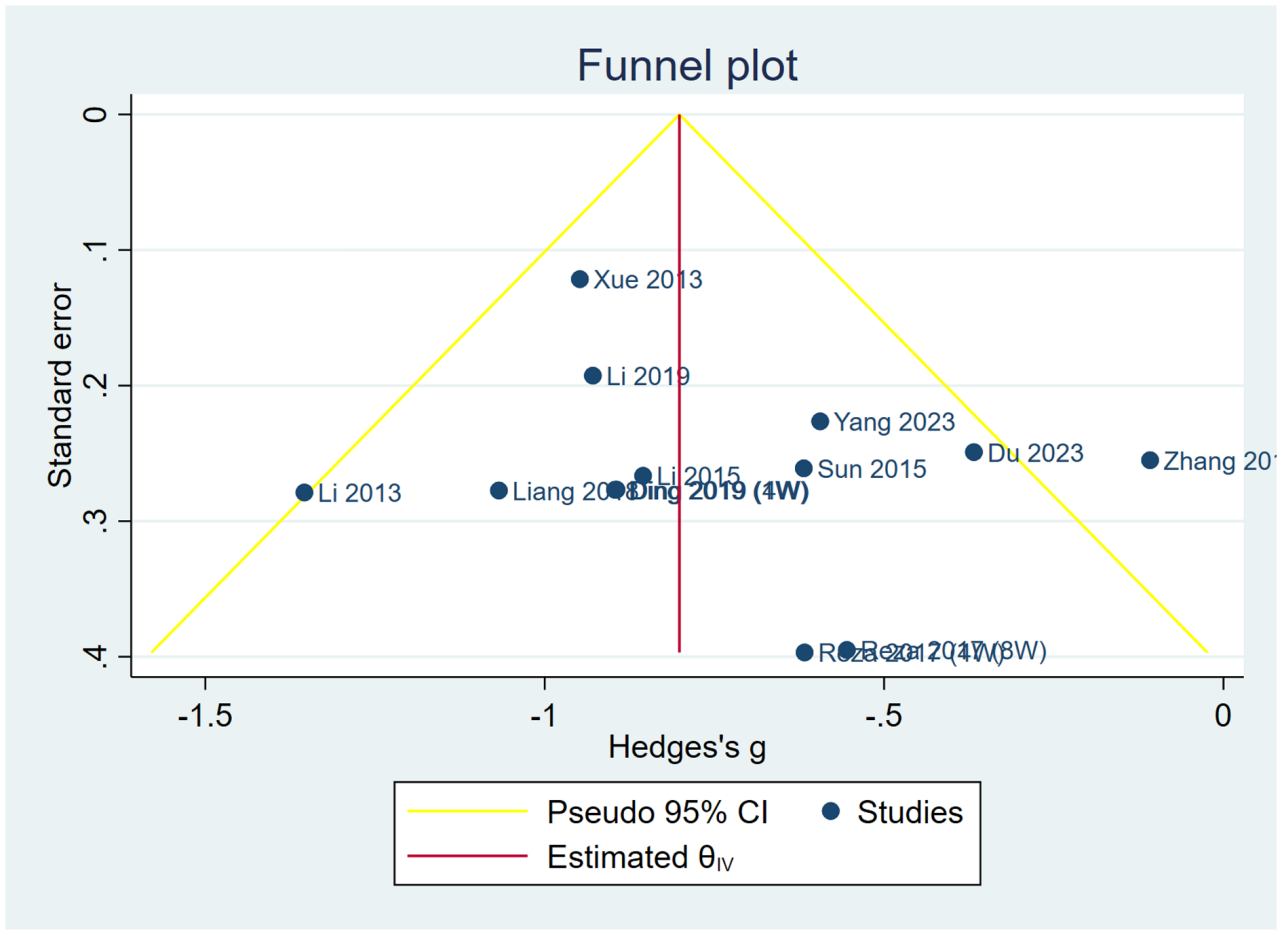
Funnel plots of SET on the VAS score.

**Figure 8:**
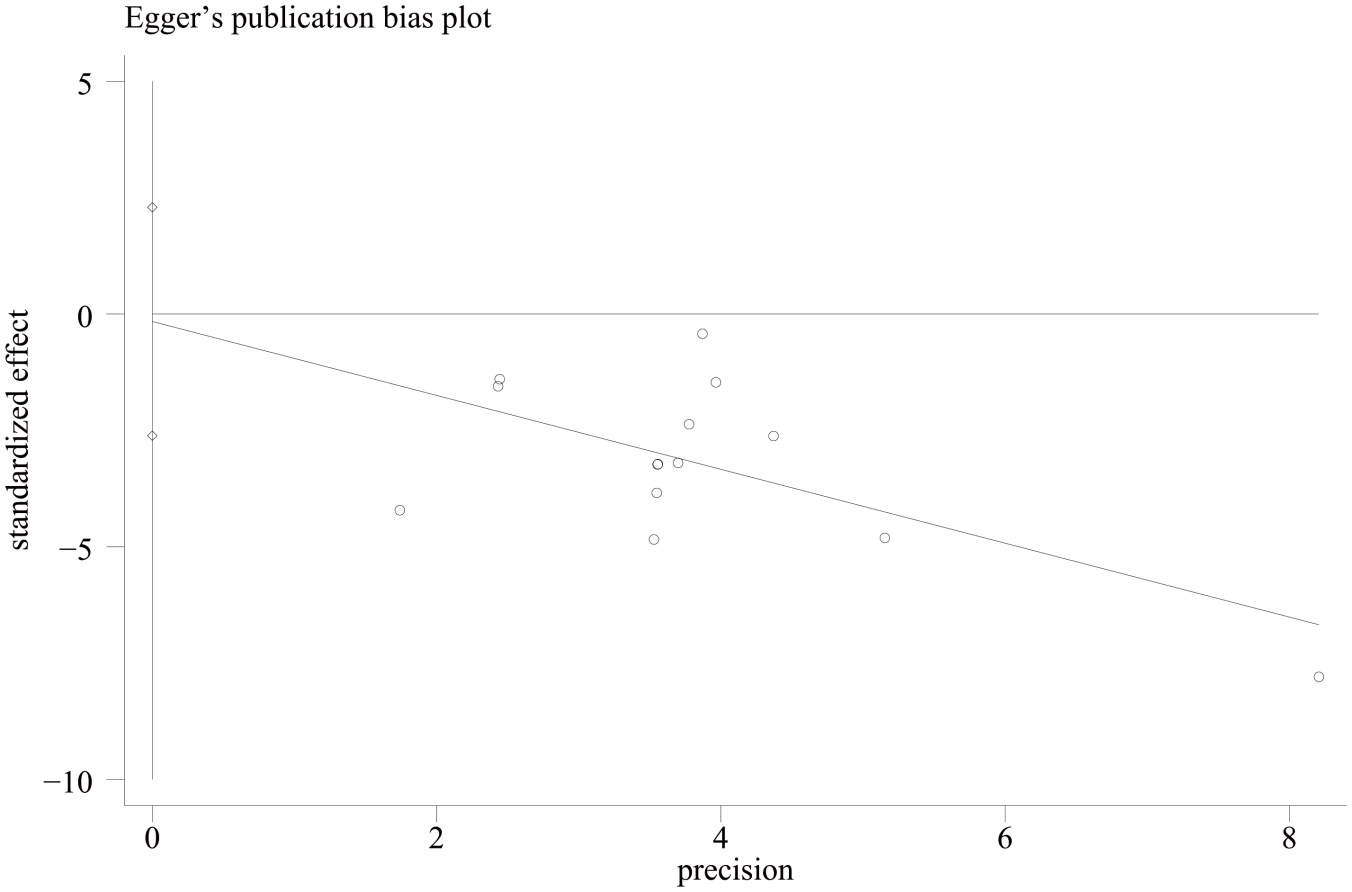
Egger’s publication bias plot for the VAS score.

Funnel plots and Egger’s asymmetry test were conducted for the VAS scores of the outcome indicators that included more than 10 studies. The results showed that the left and right sides were largely symmetrical, with the majority of the studies positioned in the upper middle. However, one study (30) fell outside the 95% confidence interval (dashed angled lines) (see Figure 7). The Egger’s test indicated no publication bias (*p* = 0.888) (see Figure 8).

## 4 Discussion

To the best of our knowledge, this is the first meta-analysis that evaluates the efficacy of suspension exercise training in the treatment of LDH, focusing particularly on its effect on patients with lumbar disk herniation. A total of 11 studies involving 943 patients with lumbar disk herniation were included in this meta-analysis. The results indicated that suspension exercise training significantly improved the VAS, JOA, and ODI scores among the patients with LDH. Subgroup analyses revealed that the combined modality of suspension exercise training was the main source of heterogeneity in the JOA scores.

The VAS score (17) is a valid scoring method for measuring pain, represented on a straight line of 10 cm length, where one end signifies 0 for no pain and the other end signifies 10 for the most severe pain. This allows testers to assess the level of pain according to their own sensations; the lower the score, the lesser the pain, while the higher the score, the greater the pain. The results of this study showed that suspension exercise training significantly reduced the VAS scores among the patients with lumbar disk herniation, which is consistent with a previous study (31). However, unlike the previous study, which showed that suspension exercise training reduced the VAS score by 4.37 points, this overview showed that SET only reduced it by 0.96 points. This difference is mainly due to the lack of a control group in the study as the VAS scores do not represent the mean difference between the experimental and control groups. Suspension exercise training can significantly reduce the VAS scores of patients with LDH for three main reasons. Firstly, suspension exercise training increases the lumbar intervertebral space by stretching the spinal column, which reduces the compression of lumbar disks on the nerves (32). Secondly, it can improve the microcirculation of lumbar soft tissues and accelerate the subsidence of inflammatory substances (33), thus reducing the production of pain factors. Finally, it can enhance the strength and coordination of the trunk muscle groups and improve the stability of the lumbar spine, thus reducing pain in patients with LDH (22).

The JOA score (18) is an effective scoring method for assessing the neurological functional status and daily living ability of patients with lumbar spine disease. It includes 25 scoring items, covering subjective symptoms, clinical symptoms, and daily living ability; the higher the score, the more obvious the functional improvement. The results of this overview showed that suspension exercise training significantly improved the JOA scores among the patients with lumbar disk herniation, which is consistent with a previous study (34). However, unlike the previous study, which showed that suspension exercise training improved the JOA scores by 5.28 points compared to traditional massage therapy, our study indicated that it only improved the scores by 3.29 points. We believe that this difference is mainly due to the intervention modality as suspension exercise training is not the only variable in the experimental and control groups. Suspension exercise training significantly improved the JOA scores among the patients with LDH. We believe that, on the one hand, suspension exercise training stimulates the neuromuscular coordination of contractions between the trunk muscles and major muscle groups of the body, thereby improving neuromuscular function (35). On the other hand, suspension exercise training enhances the stability of the spine by strengthening the strength and coordination of the trunk muscle groups, thereby improving the body’s balance, control, and stabilization during exercise (27). However, although suspension exercise training significantly improved the JOA scores among the patients with LDH, the study results showed a high degree of heterogeneity, which was significantly reduced after the deletion of one study (28). We believe this may be attributed to the intervention of suspension exercise training combined with pharmacotherapy. The subgroup analyses showed that suspension exercise training combined with traditional Chinese medicine had a better effect on improving the JOA scores among the patients with LDH. Therefore, future studies need to further validate the therapeutic effects of suspension exercise training combined with traditional Chinese medicine to determine the optimal combination modality.

The ODI score (19) is a validated scale commonly used to evaluate dysfunction related to lower back pain. There are a total of 10 scoring components, covering a variety of aspects such as pain level, daily living ability, walking, and standing, with higher scores indicating more severe dysfunction. The results of this study showed that suspension exercise training significantly reduced the ODI scores among the patients with LDH, which is consistent with a previous study (36). This is mainly due to the fact that suspension training addresses gravity with the help of adjustable slings and ropes, placing the patient on an unstable plane. This increases the stimulation of proprioceptive input in the lumbar core stabilizing muscle groups through safe, stepwise training, activates and recruits more motor units to enhance the muscle strength of target muscle groups, rebuilds the normal muscle movement pattern, and strengthens the stability of the spine, ultimately improving lumbar spine function (26) and reducing the ODI scores of patients with LDH. However, the results of the study showed a high degree of heterogeneity, which was significantly reduced after the deletion of one study (28), although there was no valid reason to delete this study. The subgroup analyses of the intervention duration and treatment modalities did not reveal a source of heterogeneity. We believe that the combined modality of suspension exercise training, the location of lumbar disk herniation, and the population affected by it might have been the sources of heterogeneity, which need to be further investigated in future studies. On the other hand, only five studies were included in the ODI scores of this review, and the small number of studies might have contributed to the high heterogeneity.

## 5 Limitations

This study has the following three limitations. First, the number of studies included and their reliability need further improvement. Second, the VAS, JOA, and ODI scores rely on subjective evaluation scales. Finally, the meta-analyses of the JOA and ODI scores showed high heterogeneity.

## 6 Conclusion

In conclusion, suspension exercise training significantly improved the VAS, JOA, and ODI scores of the patients with lumbar disk herniation; however, there was a high degree of heterogeneity in the JOA and ODI scores Further validation is needed in the future for different populations with lumbar disk herniation, the specific locations of its occurrence, and the combined modalities of suspension exercise training.

## Data Availability

The original contributions presented in the study are included in the article/Supplementary material, further inquiries can be directed to the corresponding author.
