# Supplementary material for: Effects of suspension exercise training in the treatment of lumbar disk herniation: a systematic review and meta-analysis
Source: Front Neurol. 2024 Dec 2;15:1455505. doi: 10.3389/fneur.2024.1455505 (PMC11648423; doi:10.3389/fneur.2024.1455505)
Supplement: Supplementary file 1 [file Table_1.docx]

| Step | Pubmed search strategy |
| --- | --- |
| #1 | **(((((Sling exercise[Title/Abstract]) OR (Suspension exercise[Title/Abstract])) OR (Sling training[Title/Abstract])) OR (Suspension training[Title/Abstract])) OR (Sling exercises[Title/Abstract])) OR (Suspension exercises[Title/Abstract])** |
| #2 | (((((((((((((((((((((((((((lumbar disc herniation[Title/Abstract]) OR (Lumbar intervertebral disc herniation[Title/Abstract])) OR (LDH[Title/Abstract])) OR (discal lumbosciatica[Title/Abstract])) OR (disk hernia, lumbar[Title/Abstract])) OR (hernia disci lumbalis[Title/Abstract])) OR (hernia, lumbar disk[Title/Abstract])) OR (herniated lumbar disc[Title/Abstract])) OR (herniated lumbar disk[Title/Abstract])) OR (lumbar disc hernia[Title/Abstract])) OR (lumbar disc prolapse[Title/Abstract])) OR (lumbar disc protrusion[Title/Abstract])) OR (lumbar discal hernia[Title/Abstract])) OR (lumbar discal prolapse[Title/Abstract])) OR (lumbar discal protrusion[Title/Abstract])) OR (lumbar disk herniation[Title/Abstract])) OR (lumbar disk prolapse[Title/Abstract])) OR (lumbar disk protrusion[Title/Abstract])) OR (lumbar intervertebral disc hernia[Title/Abstract])) OR (lumbar intervertebral disk hernia[Title/Abstract])) OR (lumbar intervertebral disk herniation[Title/Abstract])) OR (lumbar vertebral disc hernia[Title/Abstract])) OR (lumbar vertebral disc herniation[Title/Abstract])) OR (lumbar vertebral disk hernia[Title/Abstract])) OR (lumbar vertebral disk herniation[Title/Abstract])) OR (median lumbar disk prolapse[Title/Abstract])) OR (herniated lumbar intervertebral disc[Title/Abstract])) OR (herniated lumbar intervertebral disk[Title/Abstract]) |
| #3 | #1 AND #2 |
