# Supplementary material for: Effects of suspension exercise training in the treatment of lumbar disk herniation: a systematic review and meta-analysis
Source: Front Neurol. 2024 Dec 2;15:1455505. doi: 10.3389/fneur.2024.1455505 (PMC11648423; doi:10.3389/fneur.2024.1455505)
Supplement: Supplementary file 2 [file Table_2.docx]

Table 2 Detailed sensitivity analysis table for VAS scores.

| One study deleted | MD | 95% CI | *P* | *I^2^* |
| --- | --- | --- | --- | --- |
| Ding et al. (1W) 2019 | -0.98 | -1.18 ~ -0.79 | *P* < 0.00001 | 25 |
| Ding et al. (4W) 2019 | -0.99 | -1.18 ~ -0.79 | *P* < 0.00001 | 24 |
| Du et al. 2023 | -0.96 | -1.14 ~ -0.77 | *P* < 0.00001 | 29 |
| Liang et al. 2018 | -0.93 | -1.10 ~ -0.75 | *P* < 0.00001 | 19 |
| Li et al. 2015 | -0.93 | -1.10 ~ -0.77 | *P* < 0.00001 | 16 |
| Li et al. 2019 | -0.98 | -1.19 ~ -0.77 | *P* < 0.00001 | 28 |
| Li et al. 2013 | -0.91 | -1.18 ~ -0.74 | *P* < 0.00001 | 12 |
| Reza et al. (4W) 2017 | -0.96 | -1.15 ~ -0.77 | *P* < 0.00001 | 29 |
| Reza et al. (8W) 2017 | -0.95 | -1.14 ~ -0.77 | *P* < 0.00001 | 29 |
| Sun et al. 2015 | -0.95 | -1.14 ~ -0.76 | *P* < 0.00001 | 28 |
| Xue et al. 2023 | -0.94 | -1.16 ~ -0.72 | *P* < 0.00001 | 27 |
| Yang et al. 2023 | -0.96 | -1.16 ~ -0.77 | *P* < 0.00001 | 29 |
| Zhang et al. 2018 | -0.99 | -1.13 ~ -0.85 | *P* < 0.00001 | 0 |

Note: One study deleted indicates the combined results of the remaining studies after deletion of the study.
