# Supplementary material for: Effects of suspension exercise training in the treatment of lumbar disk herniation: a systematic review and meta-analysis
Source: Front Neurol. 2024 Dec 2;15:1455505. doi: 10.3389/fneur.2024.1455505 (PMC11648423; doi:10.3389/fneur.2024.1455505)
Supplement: Supplementary file 3 [file Table_3.docx]

Table 3 Detailed sensitivity analysis table for JOA scores.

| One study deleted | MD | 95% CI | *P* | *I^2^* |
| --- | --- | --- | --- | --- |
| Ding et al. (4W) 2019 | 3.08 | 1.38 ~ 4.78 | *P* = 0.0004 | 89 |
| Du et al. 2023 | 3.72 | 1.89 ~ 5.54 | *P* < 0.0001 | 93 |
| Li et al. 2015 | 3.22 | 1.53 ~ 4.91 | *P* = 0.0002 | 93 |
| Li et al. 2019 | 3.55 | 1.57 ~ 5.53 | *P* = 0.0004 | 93 |
| Li et al. 2013 | 2.59 | 1.09 ~ 4.10 | *P* = 0.0007 | 90 |
| Xue et al.. 2023 | 3.73 | 2.17 ~ 5.29 | *P* < 0.00001 | 83 |
| Yang et al. 2023 | 3.18 | 1.38 ~ 4.98 | *P* = 0.0005 | 92 |

Note: One study deleted indicates the combined results of the remaining studies after deletion of the study.
