# Supplementary material for: Effects of suspension exercise training in the treatment of lumbar disk herniation: a systematic review and meta-analysis
Source: Front Neurol. 2024 Dec 2;15:1455505. doi: 10.3389/fneur.2024.1455505 (PMC11648423; doi:10.3389/fneur.2024.1455505)
Supplement: Supplementary file 4 [file Table_4.docx]

Table 4 Detailed sensitivity analysis table for ODI scores .

| One study deleted | MD | 95% CI | *P* | *I^2^* |
| --- | --- | --- | --- | --- |
| Liang et al. 2018 | -5.33 | -7.71 ~ -2.94 | *P* < 0.0001 | 89 |
| Li et al. 2015 | -4.72 | -6.54 ~ -2.90 | *P* < 0.00001 | 85 |
| Xue et al. 2023 | -5.37 | -8.33 ~ -2.42 | *P* = 0.0004 | 80 |
| Yang et al. 2023 | -6.13 | -8.19 ~ -4.06 | *P* < 0.00001 | 85 |
| Zhang et al. 2018 | -6.14 | -9.09 ~ -3.20 | *P* < 0.0001 | 82 |

Note: One study deleted indicates the combined results of the remaining studies after deletion of the study.
